# Progress in Research on Antitumor Drugs and Dynamic Changes in Skeletal Muscles

**DOI:** 10.3389/fphar.2022.893333

**Published:** 2022-07-06

**Authors:** Ting Xu, Zhen-Hao Li, Ting Liu, Cai-Hong Jiang, Ya-Juan Zhang, Hui Li, Ying Jiang, Juan Zhao, Wen-Jing Guo, Jia-Yuan Guo, Lu Wang, Jia-Xuan Li, Jing Shen, Gao-Wa Jin, Ze-Wei Zhang, Quan-Fu Li

**Affiliations:** ^1^ Ordos Clinical College, Inner Mongolia Medical University, Ordos, China; ^2^ School of Public Health and Management, Wenzhou Medical University, Wenzhou, China; ^3^ Department of Medical Oncology, Ordos Central Hospital, Ordos, China; ^4^ Ordos Clinical College, Baotou Medical College, Ordos, China; ^5^ State Key Laboratory of Oncology in South China, Department of Radiation Oncology, Sun Yat-sen University Cancer Center, Collaborative Innovation Center for Cancer Medicine, Guangzhou, China

**Keywords:** sarcopenia, dynamic change, chemotherapy drugs, targeted drugs, antitumor therapy

## Abstract

**Objective:** To review the research progress of reltionship between antitumor drugs and the dynamic changes of the skeletal muscles during treatment phase.

**Background:** Sarcopenia is a common disease in patients with tumors, and it has been agreed that patients with tumors and sarcopenia experience more serious adverse reactions and have a shorter long-term survival after antitumor therapy than patients without sarcopenia. Antitumor drugs whilst beneficial for tumor regression, interferes and synergizes with cancer-induced muscle wasting/sarcopenia, induced myodemia or intramuscular fat and the two conditions often overlap making it difficult to drive conclusions. In recent years, increasing attention has been paid to the dynamic changes in skeletal muscles during antitumor drug therapy. Dynamic changes refer not only measurement skeletal muscle quantity at baseline level, but give more emphasis on the increasing or decreasing level during or end of the whole treatment course.

**Methods:** We retrievaled published English-language original research articles *via* pubmed, those studies mainly focused on repeated measurements of skeletal muscle index using computed tomography (CT) in cancer patients who received antitumor drug treatment but not received interventions that produced muscle mass change (such as exercise and nutritional interventions).

**Conclusion:** This article will summarize the research progress to date. Most of antineoplastic drug cause skeletal muscle loss during the treatment course, loss of L3 skeletal muscle index is always associated with poor clinical outcomes.

## Introduction

Sarcopenia is associated with toxicity and side effects and affects the response rate and prognosis of patients undergoing treatment for various solid tumors, on the other hand a series of treatments that are done in the presence of cancer may also affect the muscle differently (Bozzetti, 2020). In the last decade, sarcopenia has become a new research direction in tumor nutrition, mainly focusing on the relationship between the quantity and quality of skeletal muscle at baseline and the response to chemotherapy. Since 2015, attention has been paid to the relationship between the dynamic changes in skeletal muscle, the side effects and efficacy of treatment, and the prognosis of patients before and after treatment. According to a meta-analysis, 60% of studies on the changes in skeletal muscle during treatment were published after 2018 (Jang et al., 2020), and studies on sarcopenia have developed from static to dynamic monitoring, based on the needs of clinical tumor nutrition. Therefore, it is necessary to summarize the research progress on antitumor drugs and the dynamic changes in the skeletal muscles.

## Concepts Related to Changes in Skeletal Muscles

### Sarcopenia

In 1989, Rosenberg first introduced the term sarcopenia to describe a syndrome characterized by the progressive and systemic reduction in age-related skeletal muscle mass and strength (Rosenberg, 1989), but its definition is constantly being improved. In 2010, the European Working Group for Sarcopenia in Older People (EWGSOP) defined sarcopenia as a syndrome with the adverse outcomes of limb dysfunction, reduced quality of life, and death due to the progressive and extensive loss of skeletal muscle mass and strength (Cruz-Jentoft et al., 2010). The International Working Group for Sarcopenia (IWGS) released a similar definition 1 year later (Fielding et al., 2011). In 2014, the Asian Working Group for Sarcopenia (AWGS) defined sarcopenia (Chen et al., 2014), and other international academic organizations related to sarcopenia have followed by publishing their own standards (Table 1).

**TABLE 1:** Definitions of sarcopenia of different research groups.

Research group, years	Appendicular skeletal muscle mass (Cut-off value)	Physiological function (Normal gait speed)	Muscle strength (grip strength)	Summary definition
EWGSOP Cruz-Jentoft et al. (2010)	M: ≤7.26 kg/m^2^	Gait speed ≤0.8 m/s (4-mcourse)	M: <30 kg	“Sarcopenia” low lean mass plus either slowness or weakness “Severe sarcopenia” all three criteria
	F: ≤5.45 kg/m^2^		F: <20 kg	
AWGS Chen et al. (2014), Chen et al. (2020)	ALM/ht^2^	Gait speed ≤0.8 m/s (6-mcourse)	M: <26 kg	Sarcopenia as “age-related loss of muscle mass, plus low muscle strength, and/or low physical performance” and specified cut-offs for each diagnostic component
	DXA: M: <7.0 kg/m^2^		F: <18 kg	
	F: <5.4 kg/m^2^ or BIA: M: <7.0 kg/m^2^			
	F: <5.7 kg/m^2^			
IWGS Fielding et al. (2011)	ALM/ht^2^	Gait speed <1.0 m/s	Not included	Sarcopenia: both slowness and low lean mass
	M: ≤7.23 kg/m^2^			
	F: ≤5.67 kg/m^2^			
FNIH Studenski et al. (2014)	ALM/BMI	Gait speed ≤0.8 m/s	M: <26 kg	Weakness and low lean mass is the presence of low grip strength and low lean mass
	M: <0.789 kg/BMI		F: <16 kg	
	F: <0.512 kg/BMI			
SSCWD Bauer et al. (2019)	ALM/Ht^2^	Gait speed <1.0 m/s OR <400 m (6-min walk)	Not included	“Sarcopenia with limited mobility”: presence of low lean mass plus slow gait speed
	M: ≤7.26 kg/m^2^			
	F: ≤5.67 kg/m^2^			

EWGSOP, European working group on sarcopenia in older people; AWGS, Asian working group for sarcopenia; IWGS, international working group on sarcopenia; FNIH, foundation for the national institutes of health; SSCWD, society on sarcopenia, cachexia and wasting disorders; DXA, dual-energy X-ray absorpometry; BIA, bioelectrical impedance analysis; BMI, body mass index; ALM, appendicular lean mass; M, male; F, female.

In early definitions, sarcopenia only referred to lean body mass (Newman et al., 2003; Delmonico et al., 2007), but in the latest definitions, it refers to a syndrome (Table 1), i.e., a state of reduced skeletal muscle mass leading to physical dysfunction, of which skeletal muscle mass, including/excluding reductions in muscle function (muscle strength, e.g., grip strength, and body function, e.g., walking speed), is a core element in all definitions. In terms of the definitions of sarcopenia in the table above, the three factors, i.e., reduced skeletal muscle mass, reduced muscle strength, and decreased body function, should occur simultaneously for the EWGSOP and AWGS definitions. In addition to reduced skeletal muscle mass, decreased body function should occur for the IWGS and SSCWD definitions, and skeletal muscle loss and decreased muscle strength are necessary conditions for the FNIH definition, with reduced physical function as an additional condition.

### Myodemia

Muscle tissue usually contains only a small amount of fat, so excessive fat deposits are considered a pathological phenomenon called myodemia. Low muscle radiation density, which is usually represented as low mean muscle attenuation or low mean skeletal muscle density (SMD) on computed tomography (CT) images during cancer treatment, is an indicator of poor prognosis (Daly et al., 2018; Stretch et al., 2018). Myodemia is a disease different from but associated with loss of muscle mass (Amini et al., 2019). Although the specific physiological mechanism of cancer-related myodemia remains unclear; there are already theories that gene expression, cell morphology, oxidative phosphorylation, and decreased lipid oxidation may be involved in its development (Stretch et al., 2018). Aro et al. (2020) found that 108 out of 348 patients with colorectal cancer had myodemia postoperatively, and myodemia, rather than sarcopenia, was found to be an independent risk factor for a lower 5-year survival rate (*p* = 0.002). A meta-analysis conducted by Aleixo et al. (2020) showed that patients with myodemia, defined using SMD, had a higher risk of mortality when compared with those without myodemia (*p* < 0.00001). Low SMD reflects muscle fat content, suggesting that skeletal muscle fat infiltration (myodemia) is closely related to the treatment and prognosis of patients with tumors.

### Sarcopenic Obesity

In the case of aging and malignant tumors, the loss of muscle mass may be accompanied by a constant or even increased fat mass, which is defined as sarcopenic obesity (sarcopenia with BMI > 25 kg/m^2^). Concurrent sarcopenia and obesity have been associated with the worst outcomes. In a study on 548 patients with pancreatic head cancer undergoing pancreaticoduodenectomy, Ryu et al. (2020) showed that 252 patients (45.9%) had sarcopenia and 202 patients (36.9%) had sarcopenic obesity preoperatively. The 5-year survival rates of patients without sarcopenia and patients with sarcopenia were 28.4 and 23.4%, respectively (*p* = 0.046). According to a multivariate analysis, sarcopenic obesity was the only independent risk factor for postoperative complications (*p* = 0.018), which may be due to the nutritional status of patients who are overweight/obese often concealing muscle consumption (obesity paradox) (Martin, 2016) and leading to a poor prognosis. In a study on 250 patients with solid tumors in the respiratory tract or gastrointestinal tract, Prado et al. (2013) found that sarcopenic obesity was associated with poorer physical functioning and was an independent predictor of survival.

## Progress of the Third Lumbar Spine Vertebral Body Skeletal Muscle Parameters in the Diagnosis of the Sarcopenia Threshold

### Skeletal Muscle Measurements

The three most commonly used methods for measuring body composition in patients with cancer include bioelectrical impedance analysis, dual-energy X-ray absorption, and CT scan. Unlike the first two options, CT can be used to measure body composition in tissues and organs, and it is considered the gold standard for body composition assessment, accurately quantifying tissue area, volume, and attenuation value. The use of CT for body composition assessment in non-cancer populations is limited by its high levels of radiation, high cost, and low availability. However, CT is a routine method for diagnosing and monitoring the disease outcomes of patients with cancer. Therefore, it can be used to assess body composition in cancer patient population. Total skeletal muscle area (SMA) at the third lumbar spine (L3) is a measure of muscle mass using CT, as the L3 SMA is well correlated with total body muscle mass in healthy individuals (Shen et al., 2004). The muscles in the L3 region include the psoas major muscle, paraspinal muscle (erector spine and quadratus lumborum), and abdominal wall muscle (transverse abdominis, internal and external oblique, and rectus abdominis). Based on the Hounsfield unit threshold, the corresponding image analysis software, e.g., sliceOmatic (TomoVision), OsiriX DICOM viewer (Pixmeo), and ImageJ (National Institutes of Health), can be used to evaluate muscle tissue and calculate the skeletal muscle index [SMI = total SMA (cm^2^) ÷ height (m^2^)].

### Skeletal Muscle Thresholds for the Diagnosis of Sarcopenia

Finding the optimal critical value for the diagnosis of sarcopenia is one of the biggest challenges in current research. Prado et al. first proposed an applicable sarcopenia threshold, but many other research groups went on to publish their own thresholds for diagnosing sarcopenia (Table 2).

**TABLE 2:** Thresholds/cutoff values of skeletal muscle-related parameters partially defined by CT for sarcopenia in tumor patients.

Author, year of publication	Country	N.	Tumor types/stage	Sarcopenia cut-points (SMI = cm^2^/m^2^, unless Indicated otherwise)	
				Male	Female
Prado et al. (2013)	Canada	250	Respiratory and GI/I–IV*	<52.4	<38.5
van Vledder et al. (2012)	Netherlands	196	Colorectal cancer with liver metastasis	<43.75	<41.1
Martin et al. (2013)	Canada	1,473	Lung and GI/I–IV	<43·0 (BMI < 25 kg/m^2^)	<41.0
				<53·0 (BMI ≥ 25 kg/m^2^)	
Lanic et al. (2014)	France	82	Diffuse large B cell lymphoma	<55.8	<38.9
Fujiwara et al., (2015)	Japan	1,257	Hepatocellular carcinoma/I–IV	<36.2	<29.6
Kimura et al. (2015)	Japan	134	Lung cancer/III–IV	<41.0	<38·0
Choi et al. (2015)	Korea	484	Pancreatic cancer/advanced	<42.2	<33.9
Zhuang et al. (2016)	China	937	Undergoing gastrectomy for GC	<40.8	<34.9
Caan et al. (2017)	USA	3,262	Colorectal cancer/I–III	<52.3 (BMI < 30 kg/m^2^)	<38.6 (BMI < 30 kg/m^2^)
				<54.3 (BMI ≥ 30 kg/m^2^)	<46.6 (BMI ≥ 30 kg/m^2^)

*Body-mass index (BMI) ≥ 30 kg/m^2^

As shown in Table 2, there is extensive variability in the threshold values for musculoskeletal-related parameters used to define sarcopenia in these studies, which may be due to the substantial operational differences between the different definitions, e.g., the SMI assessment methods, and standardized methods. In addition, the race, age, gender, disease type, disease stage, treatment method, physical activity, and degree of obesity of the subjects can also affect their muscle condition (Heymsfield et al., 2015). For example, the threshold published by Prado et al. (2013) was derived from a cohort of patients with obesity and cancer, which may have limited applicability in patients without obesity. Therefore, it will be of great practical significance to actively explore the muscle threshold of the L3 vertebral body suitable for diagnosing sarcopenia in patients with tumors in this region.

Although CT images can be used to accurately assess muscle mass, there is no standardized and feasible method to measure muscle function. Therefore, studies on sarcopenia in patients with cancer can only partially define sarcopenia.

## Dynamic Changes in Skeletal Muscle During Antineoplastic Drug Therapy

### Dynamic Changes in Skeletal Muscles During Chemotherapy

The progressive loss of muscle tissue during chemotherapy may, in part, be due to uncontrolled muscle proteolysis aggravated by tumor growth (Prado et al., 2013). Some adverse events during chemotherapy, such as fatigue, loss of appetite, nausea, vomiting, and diarrhea, will adversely affect food intake and physical activity, further leading to a sharp decline in muscle mass (Kakinuma et al., 2018). Tumour implantation and cisplatin induced muscle atrophy by activating pro-inflammatory cytokines, p38-C/EBP-β, and myostatin, and by downregulating Akt, myoD, and myogenin, leading to activation of ubiquitin-proteasome-mediated proteolysis and muscle weakness. In addition, chemotherapy is likely to cause mitochondrial damage, reducing the cytochrome complex and peroxisome proliferator-activated receptor required for oxidative phosphorylation, thereby affecting energy metabolism, mitochondrial biopotency, and muscle fiber metabolism (Chen et al., 2015). Chemotherapy may also increase reactive oxygen species and induce oxidative stress in muscles (Chen et al., 2015). Cisplatin induces Nf-KB expression which targets not only muscle fibers but also muscle stem cells may induces muscle wasting, and doxorubicin cause muscle atrophy through increase the Trim63, Fbxo32, Myostatin, FoxO mRNA expression levels of the proteolytic pathways in gastrocnemius muscle, interestingly exercise could counteracts cachexia and sarcopenia through the mechanisms include autophagic flux regulation, the reduction of Pax7 expression in satellite cells, and the release of Hsp60 from muscle cells (Coletti, 2018; Damrauer et al., 2018; Alves de Lima et al., 2020).

Tumor growth factor beta protein (TGF-β) will increase with chemotherapy and upregulate myostatin, thus affecting the balance of muscle catabolism (Chen et al., 2015; Chen et al., 2016). Chemotherapy will also lead to reduced muscle microvessels through anti-angiogenesis (Barreto et al., 2016). In conclusion, chemotherapy as one of the main antitumor treatment methods, it may cause or aggravate muscle loss in patients due to drug-related adverse reactions or other complexed mechabism, and there is a close relationship between them.

### Palliative Chemotherapy

In a longitudinal study on changes in skeletal muscle mass in 70 patients with advanced squamous cell lung cancer, Lee et al. found that the mean percentage loss in skeletal muscle area was 16.5 ± 11.0% between the first and last CT scans, and the mean decrease in muscle area was 17.28 ± 13.00 cm^2^ (2.64 ± 2.36 cm^2^/month). Skeletal muscle loss accelerated over time and peaked in the last 3 months of the disease (*p* < 0.001). In addition, patients with skeletal muscle loss ≥3.24 cm^2^/month had a shorter overall survival (OS) than those with a slower rate of loss (*p* < 0.001) (Lee et al., 2021a). In a study on 111 patients who received first-line palliative chemotherapy for advanced gastric cancer, Park et al. (2020) found that the median SMI value decreased by 4.0 cm^2^/m^2^, 4.5 cm^2^/m^2^, and 3.8 cm^2^/m^2^ in patients got objective response, stable and disease progressive, respectively. Skeletal muscle mass decreased significantly during chemotherapy, but it was not associated with the efficacy of chemotherapy. According to a multivariate analysis, sarcopenia at baseline (*p* = 0.021) and reduced SMI during chemotherapy (*p* = 0.032) constituted significant adverse prognostic factors for the survival rate.

In a study with pancreatic cancer patients, Choi et al. (2015) found that 21.3% of patients (103 out of 484) were diagnosed sarcopenia at baseline. SMI decreased in 60.9% of male patients and 40.6% of female patients during chemotherapy. BMI decreased in 37.3% of patients, with no gender-related differences. Based on the multivariate analysis, increased sarcopenia (*p* < 0.001), decreased BMI (*p* = 0.002), and SMI (*p* = 0.004) during chemotherapy were adverse prognostic factors for OS. The OS of male patients was affected by increased sarcopenia (*p* < 0.001) and decreased SMI (*p* = 0.001), and the OS of female patients was affected by being overweight at diagnosis (*p* = 0.006), decreased BMI (*p* = 0.032), and SMI (*p* = 0.014) during chemotherapy. The change of BMI in patients with stable SMI during chemotherapy had no effect on OS (*p* = 0.750), but the decreased in SMI in patients with stable BMI was correlated with decreased in OS (*p* = 0.002). Cho et al. (2017) revealed that low skeletal muscle mass at diagnosis (*p* = 0.008) and decreased SMI during chemotherapy (*p* < 0.001) were adverse prognostic factors for OS in 524 patients with cholangiocarcinoma (CCA). A subgroup analysis showed that patients who were overweight or obese (BMI ≥ 25 kg/m^2^) and had low skeletal muscle mass had a worse OS (*p* < 0.001). In addition, patients with reduced BMI and SMI during chemotherapy were found to have a shorter OS (*p* < 0.001). Regardless of BMI changes during chemotherapy, patients with reduced SMI had a shorter survival, and a decrease in BMI had no effect on the survival of patients with stable SMI (*p* = 0.576). However, a clinical study conducted by Dijksterhuis et al. (2019) on patients with advanced esophageal cancer undergoing palliative chemotherapy (*n* = 88) yielded different results: muscle loss (48.9%) and low muscle density (50.0%) occurred in approximately half of the patients, but low SMI, low SMD, muscle loss before treatment, sarcopenic obesity, and changes in SMI during chemotherapy were not associated with progression-free survival (PFS) or OS and treatment-related toxicity. These inconsistencies could be explained by geographic differences in the study samples and different sample sizes, chemotherapy drugs, and cancer types, and all these studies showed a reduction in skeletal muscle mass during chemotherapy.

Interestingly, in a study on a small sample of patients with advanced lung cancer (*n* = 35), Stene et al. (2015) showed that almost half the patients (*n* = 16) had stable or increased muscle mass during chemotherapy without any cachexia therapy, 14 patients (56%) responded well to chemotherapy and two patients showed progressive disease (*p* = 0.071). When compared with the muscle loss group, patients with stable or increased muscle mass had a significantly longer median survival (5.8 and 10.7 months, respectively; *p* = 0.073). This could suggest a greater likelihood of controlling or reversing muscle loss in patients who respond well to chemotherapy. Several studies mentioned above also showed that change in muscle mass during chemotherapy is an important prognostic factor for survival, suggesting that in the future, we should focus on the dynamic changes in muscle mass during chemotherapy rather than only measuring muscle mass prior to chemotherapy.

### Adjuvant Chemotherapy

Elliott et al. (2017) conducted a study on 252 patients with esophageal cancer who received neoadjuvant chemotherapy (NAC) and continually measured muscle mass after diagnosis, before surgery, and 1 year after surgery. The results showed that there was a significant decrease in LBM during treatment (*p* < 0.0001), and while the proportion of patients with sarcopenia increased from 15.9% at baseline to 30.8% before surgery (*p* = 0.02), baseline muscle loss was not significantly associated with disease progression (*p* = 0.41). However, muscle loss after treatment was associated with disease progression (*p* = 0.001) and surgical intolerance (*p* = 0.005). Sarcopenia was associated with reduced disease-specific survival in all patients who received treatment (*p* = 0.0002). Patients who underwent surgery after NAC had a further loss of LBM 1 year later (baseline: 58.0 ± 10.3 kg, before surgery: 55.9 ± 10.1 kg, and 1 year after surgery: 52.7 ± 9.3 kg; *p* < 0.0001) and an increased prevalence of muscle loss (baseline: 6.9%, before surgery: 21.1%, and 1 year after surgery: 34.7%; *p* < 0.0001). Another NAC study conducted by the same team on patients with esophageal cancer showed that although there was no significant change in body weight, body function, or activity habits, subjects still experienced a decrease in muscle mass and strength as a result of chemotherapy. This mainly manifested as a decrease in SMI of 5.6 cm^2^/m^2^, decrease in LBM of 4.9 kg, and a decrease in mean grip strength of 4.3 kg, and the proportion of patients with sarcopenia increased from 7 to 22% (Guinan et al., 2018). Both prospective studies showed that adjuvant chemotherapy can reduce skeletal muscle mass/strength.

In a study of 100 patients diagnosed with resectable pancreatic cancer in 2012–2015 (available baseline examination and post-treatment CT body composition data from 67 patients), Griffin et al. (2019) showed that the presence at diagnosis of cancer cachexia (*p* = 0.447) or sarcopenia (*p* = 0.497) had no effect on the ultimate resectability of the pancreatic cancer or the survival rate. Lean tissue loss (SMI, fat-free mass, and skeletal muscle mass) occurred in 73% of patients during treatment and was associated with an increased risk of death, especially fat-free mass (*p* = 0.003) and skeletal muscle mass (*p* = 0.001). Lee et al. conducted a retrospective study to explore the effect of skeletal muscle loss on the response of breast cancer to NAC. A total of 246 patients were enrolled and followed up for an average of 28.85 months. The patients with a complete or partial response were included in the response group (*n* = 208), and the rest were included in the nonresponse group (*n* = 38). The results showed that skeletal muscle loss occurred in a total of 50 patients (20.3%) after NAC compared with 30 patients (12.2%) before NAC. Skeletal muscle loss was more common in patients in the nonresponse group than in the other two groups (before NAC: 23.7 and 10.1%, respectively; *p* = 0.019 and after NAC: 55.3 and 5.8%, respectively; *p* < 0.001). According to the multivariate analysis, skeletal muscle loss before and after NAC was associated with treatment response. In the advanced stage of the disease, gradual loss of skeletal muscle was significantly associated with a poor NAC response (*p* = 0.007) (Lee et al., 2021b). In a study on NAC for esophageal cancer, Kita et al. showed that in 87 patients, SMI decreased from 45.8 cm^2^/m^2^ before treatment to 43.7 cm^2^/m^2^ after treatment (*p* = 0.092). In addition, patients with severely low SMI had a worse prognosis after chemotherapy than patients with a moderately low SMI (*p* = 0.194) (Kita et al., 2021).

In a cohort study investigating the association between skeletal muscle loss over time and the long-term survival of patients with known sarcopenia, a CT scan was done before and 2 years after surgery and long-term follow-up was done with a total of 667 patients with stage I–III colorectal cancer with no recurrence within 2 years. The patients’ body composition was analyzed at two time points. Hopkins et al. (2019) showed that sarcopenia occurred in 170 patients at the time of diagnosis, 199 patients at the end of the follow-up period, and 134 patients at both time points, i.e., sarcopenia was found in 36 patients at diagnosis but disappeared by the end of the follow-up period. A univariate analysis showed that muscle loss and skeletal muscle loss were associated with poorer OS over time. The multivariate analysis revealed that sarcopenia (*p* = 0.013) and skeletal muscle loss (*p* = 0.044) at diagnosis were two independent predictors for the worst OS. The OS was significantly worse in the presence of both sarcopenia and myodemia (*p* = 0.007).

A common finding of the above mentioned studies is the negative relationship between adjuvant chemotherapy and skeletal muscle changes. Therefore, attention should be paid to changes in muscle mass and strength, and early intervention is required even if body weight or muscle function is normal. Therefore, the accurate measurement of muscle loss to guide treatment and various interventions to delay muscle loss will improve a patient’s response to adjuvant chemotherapy and affect the clinical outcome.

### Dynamic Changes in Skeletal Muscles During Targeted Drug Therapy

Some targeted drugs are associated with sarcopenia, which may affect the efficacy of targeted therapy (Bodine et al., 2001; Antoun et al., 2010; Bai et al., 2011; Ito et al., 2013; Pirinen et al., 2014; Moryoussef et al., 2015; Gyawali et al., 2016; Barreiro and Gea, 2018). For example, the target enzymes of the most widely used tyrosine kinase inhibitors are usually responsible for activating several intracellular molecular pathways involved in tumor cell proliferation, such as phosphatidylinositol 3-kinase (PI3K), serine/threonine-specific protein kinase (Akt), and mammalian target of rapamycin (mTOR). The PI3K-Akt-mTOR pathway plays a key role in muscle protein synthesis, and the activation of the Akt-mTOR pathway and its downstream targets is crucial for regulating skeletal muscle size (Bodine et al., 2001). In contrast, poly (ADP-ribose) polymerase inhibitors can reduce muscle oxidative stress, reduce muscle catabolism, enhance muscle metabolism, improve mitochondrial function (Pirinen et al., 2014; Barreiro and Gea, 2018), and improve motor ability by enhancing mitochondrial respiration (Pirinen et al., 2014), thereby improving skeletal muscle quality.

In a study conducted on the use of sorafenib in patients with advanced kidney cancer, Antoun et al. (2010) showed that only 5% of patients were underweight at baseline. After treatment, sarcopenia occurred in 52.5% of patients, which was comprised of 72% of patients with BMI < 25 kg/m^2^ and 34% of patients with BMI > 25 kg/m^2^. The body weight of patients in the placebo group remained stable (0.8 ± 0.7 kg) over 6 months, and there was no significant change in muscle or fat mass. The body weight of patients treated with sorafenib decreased by 2.1 ± 0.6 kg at 6 months (*p* < 0.01) and 4.2 ± 0.7 kg at 12 months (*p* < 0.01), and the skeletal muscle mass decreased by 4.9% at 6 months (*p* < 0.01) and 8.0% at 12 months (*p* < 0.01). After 12 months of treatment, 71% of patients met the criteria for sarcopenia. In a study on the effect of the long-term use of mTOR inhibitors on muscle mass, 20 patients met the inclusion criteria and underwent CT scans for 14.4 ± 2.0 months, and Gyawali et al. (2016) showed that 60% of patients (12 out of 20) had sarcopenia at baseline, which increased to 75% (15 out of 20) after treatment. After the use of mTOR inhibitors for 6 months, the skeletal muscle area (*p* = 0.011) and lean body weight (*p* = 0.007) were significantly reduced, with a skeletal muscle loss rate of 2.6 cm^2^/m^2^, equivalent to a weight loss of 2.3 kg. However, there were no significant differences in adipose tissue (*p* = 0.163) and body weight (*p* = 0.262). When compared with their baseline levels, 16 patients exhibited a decrease in SMI and LBM, and nine patients gained weight, of which 7 experienced muscle loss as they gained weight.

In contrast, some studies have demonstrated the protective effect of targeted drugs on muscle: Of patients with cholangiocarcinoma undergoing treatment with selumetinib, a mitogen-activated protein kinase inhibitor, 80% experienced muscle mass and weight gain and 12% exhibited an objective response to treatment, demonstrating the benefits of selumetinib for skeletal muscle anabolism in addition to its anticancer activity (Bai et al., 2011). Imatinib mesylate can inhibit tyrosine kinase receptors, including platelet-derived growth factor receptor (PDGFR)-α. The expression of PDGFR-α in muscle interstitial progenitor cells will be simultaneously inhibited when the drug induces muscle fibrosis (Ito et al., 2013), thereby preventing or delaying muscle fibrosis. In addition, imatinib has been found to reverse sarcopenia associated with gastrointestinal stromal tumor (Moryoussef et al., 2015). In addition to the mechanism of action of the drug, the reason for the occurrence of these phenomena may be that targeted drugs mainly act on tumor-specific expression molecules or specific complexes in tumor cell signaling pathways, so targeted therapy is more effective and less toxic than chemotherapy, and thus has less impact on muscle.

### Dynamic Changes in Skeletal Muscles During Immunotherapy

Immune checkpoint inhibitors (ICIs) can enhance antitumor immunity by blocking negative regulators activated by T cells, thereby boosting the ability of the host immune system to attack cancer cells. Therefore, the effectiveness of ICIs is heavily dependent upon the host immune system, and body composition has been confirmed to be closely associated with the host immune system (Shimizu et al., 2020).

In a retrospective study on 84 patients with metastatic melanoma treated with ipilimumab (Ipi), Daly et al., (2017) evaluated the body composition using CT at baseline and after four cycles of Ipi. The results showed that 24% of patients (20 out of 84) were diagnosed with sarcopenia at baseline. A longitudinal analysis of the patients using body composition CT values available before and after four cycles of Ipi (*n* = 59) showed there were significant reductions in all body composition parameters, including SMI, SMA, whole body fat-free mass, fat mass, and muscle attenuation. SMA decreased by 3.3%/100 days (*p* ≤ 0.001), and when compared with baseline, the prevalence of sarcopenia increased from 17 to 32%. When compared with patients with a muscle loss <7.5%/100 days, patients with a muscle loss ≥7.5%/100 days had a significantly lower OS.

There are few studies on the dynamic change in skeletal muscle during immunotherapy, but some studies have shown that sarcopenia diagnosed at baseline is associated with poorer treatment response and prognosis. In a meta-analysis of 740 patients with advanced cancer treated with ICIs, Deng et al. (2021) showed that patients with sarcopenia tend to have a lower response rate than patients without sarcopenia (*p* = 0.095). PFS (*p* < 0.001) and OS (*p* < 0.001) in patients with sarcopenia were significantly lower than in those without sarcopenia. Sarcopenia was an independent adverse prognostic factor for PFS (*p* < 0.001) and OS (*p* < 0.001) in patients with advanced cancer undergoing treatment with ICIs. Similarly, in a meta-analysis, Wang et al. (2020) showed that sarcopenia diagnosed before immunotherapy was significantly associated with poorer OS and PFS, and the development or worsening of sarcopenia during treatment also predicted a decrease in OS and PFS. These two factors jointly led to a poorer disease control rate. In a retrospective study, Shiroyama et al. (2019) showed that among 42 patients with advanced non-small-cell lung carcinoma undergoing treatment with nivolumab or pembrolizumab, the prevalence of sarcopenia was 52.4%, which was significantly associated with a poor PFS (*p* = 0.004), and patients without sarcopenia had a higher overall response rate than those with sarcopenia (40.0 and 9.1%, respectively).

According to the abovementioned studies, sarcopenia can significantly reduce the efficacy of ICIs, and it is also a significant adverse predictor of survival in patients with advanced cancer undergoing treatment with ICIs. Therefore, the routine assessment of sarcopenia can help in identifying patients who may benefit from treatment with ICIs and those at high risk of disease progression, and it is highly significant for guiding clinical treatment.

## Conclusion and Outlook

Skeletal muscle loss during antineoplastic therapy is associated with adverse clinical outcomes, such as poor chemotherapy tolerance, chemotherapy efficacy, and prognosis. There is evidence that patients with sarcopenia maintain the potential for synthetic muscle metabolism, reduced muscle loss, and even muscle gain during treatment (Bai et al., 2011; Ito et al., 2013; Moryoussef et al., 2015). Therefore, multiple interventions, including nutritional support, physical exercise, and drug intervention such as Anamorelin (ONO‐7643) an high‐affinity, selective agonist of the ghrelin receptor which could significantly increased LBM and improved anorexia symptoms and the nutritional state are essential for the treatment of the loss of muscle mass or strength and sarcopenia (Aversa et al., 2017). Further oncology-specifific interventions with more robust study designs are needed to facilitate improve opportunities related to the role of nutrition in preventing and reversing low MM in cancer (Prado et al., 2020).
